# Differential Expression of Circular RNAs in Polytocous and Monotocous Uterus during the Reproductive Cycle of Sheep

**DOI:** 10.3390/ani9100797

**Published:** 2019-10-14

**Authors:** Yongfu La, Jishun Tang, Ran Di, Xiangyu Wang, Qiuyue Liu, Liping Zhang, Xiaosheng Zhang, Jinlong Zhang, Wenping Hu, Mingxing Chu

**Affiliations:** 1 Key Laboratory of Animal Genetics, Breeding and Reproduction of Ministry of Agriculture and Rural Affairs, Institute of Animal Science, Chinese Academy of Agricultural Sciences, Beijing 100193, China; layongfu@yeah.net (Y.L.); tjs157@163.com (J.T.); dirangirl@163.com (R.D.); xiangyu_wiggle@163.com (X.W.); liuqiuyue@caas.cn (Q.L.); 2 College of Animal Science and Technology, Gansu Agricultural University, Lanzhou 730070, China; Zhanglp512@163.com; 3 Institute of Animal Husbandry and Veterinary Medicine, Anhui Academy of Agricultural Sciences, Hefei 230031, China; 4 Tianjin Institute of Animal Sciences, Tianjin 300381, China; zhangxs0221@126.com (X.Z.); jlzhang1010@163.com (J.Z.)

**Keywords:** sheep, circRNAs, prolificacy, RNA-Seq, uterus

## Abstract

**Simple Summary:**

The uterus is an important reproductive organ that provides nutrition and place for embryonic development. In this study, we identified circular RNAs by deep sequencing and analyzed their expression in the uteri of polytocous and monotocous sheep (FecB++) during follicular and luteal phases. Gene Ontology (GO) and KEGG enrichment analyses revealed that the source genes of these differential circular RNAs (circRNAs) were mainly enriched in reproductive hormone- and energy metabolism-related pathways. These results provide information on the molecular mechanisms of sheep prolificacy.

**Abstract:**

CircRNA plays important roles in cell proliferation, differentiation, autophagy and apoptosis during development. However, there are few reports on circRNAs related to livestock reproduction. In this study, we identified circRNAs by deep sequencing and analyzed their expression in the uteri of polytocous and monotocous sheep (FecB++) during follicular and luteal phases. There were 147 and 364 circRNAs with differential expression in the follicular and luteal phases, respectively. GO and KEGG enrichment analysis was performed for the host genes of the circRNAs to predict the functions of differentially expressed circRNAs. These source genes were mainly involved in the estrogen signaling pathway, TGFβ signaling pathway, GnRH signaling pathway, oxytocin signaling pathway, pentose phosphate pathway, and starch and sucrose metabolism related to reproduction and energy metabolism. CircRNA expression patterns were validated by RT-qPCR. Our findings provide a solid foundation for the identification and characterization of key important circRNAs involved in reproduction.

## 1. Introduction

Small Tail Han sheep are a Chinese indigenous breed that has attracted a lot of attention because of their high prolificacy and year-round estrus [1]. In Booroola and Small Tail Han ewes, the effect of the *FecB* (*BMPR1B*) gene increased the ovulation rate and was partially dominant for litter size [2,3]. According to previously published literature, the FecB mutation has an additive effect on enhancement of the ovine ovulation number and litter size, such that one copy of the FecB mutation may increase the ovulation number by 1.5 and the litter size by 1, and two copies by 3 and 1.5, respectively [4,5,6]. However, some Small Tail Han sheep without the FecB mutation (FecB++) also had high fecundity and maintained stable heredity in production. Therefore, we speculated that, besides the FecB mutation effect, other genetic factors may also influence the litter size of Small Tail Han sheep.

Mammalian reproductive processes include ovarian follicle development, ovulation, luteinization, luteolysis, and remodeling of the endometrium [7,8]. Presently, studies of fecundity have mainly concentrated on the ovary [9,10], but the function of the uterus is also important for prolificacy. The uterus is an organ composed of endometrial and myometrial linings [11,12]. According to the steroid-induced reproductive cycle, the endometrium regenerates and differentiates during the follicular phase, and the metabolic requirements of the endometrium change during the luteal phase [13]. Embryos are supported by maternal secretions from the uterus during the preimplantation period. These secretions form a complex environment that contains proteins, amino acids, pyruvate, lactate, carbohydrates and fatty acids [14,15,16,17]. In addition, hormones [18], homeobox proteins [19], morphogens and other factors directly act on the endometrium [20] and regulated endometrial functions promote interactions between the endometrium and embryo [21]. These functions make it an important source of valuable prolificacy genes to improve sheep fecundity. Therefore, an in-depth understanding of the molecular mechanisms of uterine-related functions is important to study the reproductive features of female sheep.

Circular RNAs (circRNAs) are a unique class of non-coding RNAs originally discovered in viruses, which can be generated by direct ligation of the 5’ and 3’ ends of linear RNAs [22,23,24]. To date, RNAseq were widely used in animals and plants research [25,26,27], and thousands of circRNAs have been identified [28]. Although the functions of animal circRNAs are still being elucidated, some reports have shown that circRNAs act as a sponge for miRNAs [29], regulate gene transcription, and regulate mRNA stability [30]. For example, circRNA-9119 reduces the levels of miR-26a by acting as a microRNA sponge, thereby modulating endometrial receptivity in dairy goats [31]. With the breakthroughs of high-throughput deep sequencing technology, some circRNAs have been identified in the sheep pituitary gland [32], muscle [33,34], spleen [35] and other tissues. However, there are very few reported functions of circRNAs in the sheep reproductive system.

To date, our knowledge of the expression status of the sheep uterus is very limited. To investigate the relationship between circRNA and sheep uterine development and prolificacy, we performed total RNA-Seq for ribose depletion to reveal circRNA expression profiles of polytocous and monotocous Small Tail Han sheep. We also investigated the genomic features, length distribution, and other features of circRNA in the uterus. By comparing the circRNA expression profiles from the uteri of polytocous and monotocous Small Tail Han sheep, we identified differentially expressed circRNAs. We then performed Gene Ontology (GO) annotation and KEGG enrichment analysis, which were common analytical methods [36,37,38,39], for host genes of the differentially expressed circRNAs. CircRNA expression patterns were extensively validated by RT-qPCR. Our study provides the circRNA expression profiles of the polytocous and monotocous uteri, which may facilitate better understanding of the roles of circRNAs in the reproductive mechanisms of sheep.

## 2. Materials and Methods

### 2.1. Samples

All experiments were performed following the relevant guidelines and regulations set by the Ministry of Agriculture of the People’s Republic of China. Ethical approval was provided by the Animal Ethics Committee of IAS-CAAS (No. IASCAAS-AE-03, 12 December 2016).

Based on a TaqMan assay using the FecB mutation probe, pluriparous ewes with the FecB++ genotype (pluriparous ewes without the FecB mutation) were selected from nuclear herds of Small Tail Han sheep in the southwest region of Shandong Province, China. The estrus cycles of all experimental ewes were synchronized. A controlled internal drug release (CIDR) device (300 mg progesterone) was inserted into their vagina for 12 days. Six days after CIDR removal, the ovulation rate was detected by a laparoscopy procedure. The ovulation rate was equal to the total number of corpus lutea on both sides of a ewe’s ovary. According to the litter size and ovulation rate, 12 Small Tail Han ewes were selected and equally divided into polytocous ewes (PG, litter size and ovulation number ≥ 2) and monotocous ewes (MG, litter size and ovulation number = 1). There was no significant difference in phenotypic indexes, including average age, average weight, body length, and chest circumference, between the polytocous and monotocous ewes.

Estrus synchronization, as described above, was conducted again to collect uterine samples from six polytocous ewes and six monotocous ewes. Three polytocous ewes and three monotocous ewes were euthanized at 45–48 h after CIDR removal (follicular stage) and the other three polytocous ewes and three monotocous ewes were euthanized at 9 days after CIDR removal (luteal stage). Uterine tissues from polytocous group ewes at follicular and luteal stages were named PF (*n* = 3) and PL (*n* = 3) respectively, and the uterine tissues from monotocous group ewes at follicular and luteal stages were named MF (*n* = 3) and ML (*n* = 3), respectively. The whole fresh uterus from each ewe was collected and then washed with buffer. Uterine tissues were cut to small pieces, pulverized, and mixed with liquid nitrogen, and then stored at −80 °C until use.

### 2.2. RNA Extraction, Library Construction, and RNA-Seq

Total RNA was extracted from the uterine tissues of 12 ewes. Then, measurements of the RNA purity, concentration and integrity were conducted by 1% agarose electrophoresis, a Kaiao K5500 spectrophotometer (Beijing Kaiao Technology Development Co., Ltd., Beijing, China) and RNA Nano 6000 Assay Kit of the Agilent Bioanalyzer 2100 System (Agilent Technologies, Santa Clara, CA, USA), respectively.

Library construction was performed using approximately 3 µg total RNA from each sample. Ribosomal RNAs (rRNAs) were removed from the total RNA using a Ribo-Zero™ Gold Kit (Epicentre, Madison, WI, USA). RNA libraries of 12 samples were generated using a NEB Next Ultra Directional RNA LibraryPrep Kit for Illumina (NEB, Ipswich, MA, USA), following the manufacturer’s instructions. CircRNAs were randomly fragmented and reverse transcribed into cDNA with random primers. Second-strand cDNA was synthesized using DNA polymerase I, RNase H, dNTPs, and buffer, and the cDNA fragment was purified by QiaQuick PCR, repaired at the end, tagged was a poly(A), and ligated into Illumina sequencing adapters. The samples were amplified by PCR and sequenced in the PE150 sequencing mode (Illumina, San Diego, CA, USA) using the Illumina X-Ten platform. Raw data of the RNA-Seq have been deposited in the SRA public database (Accession number: SRP173986).

### 2.3. Sequence Mapping and Circular RNAs (circRNA) Prediction

Clean reads were mapped to the reference genome (Oar_v3.1) by the HiSAT2 alignment method. CIRI is an efficient and fast tool to identify circRNAs [40]. To ensure the reliability of other circRNAs, the BWA-MEM algorithm was used to perform a sequence splitting comparison, and then the SAM file was scanned to find PCC (paired chiastic clipping) and PEM (paired-end mapping) sites, and GT-AG splicing signals [41]. Finally, the sequence with the junction site was re-aligned with the dynamic programming algorithm. CircRNAs were blasted against the circBase for annotation. The circRNAs that could not be annotated were defined as novel circRNAs. Statistical analysis of the type, chromosome distribution and length distribution of the identified circRNA was conducted.

### 2.4. Differential Expression Analysis of circRNAs

We used SRPBM (spliced reads per billion mapping) to estimate the expression of circular RNA [42]. To identify differentially expressed circRNAs across groups, the DEseq2 package was used [43]. Since the population we selected are the same sheep breed and comes from the same farm, the growth traits are the same. In addition, all individuals are FecB++ genotype, but the litter size has difference. So, we identified circRNAs with a fold change of >1.5 and a *p*-value of <0.05 between two groups as differentially expressed circRNAs.

### 2.5. Bioinformatics Analysis

KEGG pathway annotations of the host genes for differentially expressed circRNAs were determined with the KEGG database (http://www.genome.jp). GO enrichment analysis of the host genes for differentially expressed circRNAs was performed with the GOseq R package [44]. GO and KEGG pathways with *p* < 0.05 were considered as significant, *p* < 0.05 was considered to be significant for functional enrichment. Interactions between circRNAs and miRNAs were predicted based on sequence data from miRanda (3.3a) [45].

### 2.6. Validation of the Expression of circRNAs

We randomly selected six DE-circRNAs (fold change > 1.5, *p* < 0.05), for subsequent validation. We designed primers encompassing circRNA-specific, back-splice junctions for each candidate circRNA. Details of the primer sequences are summarized in Table 1. qRT-PCR was performed using SYBR Green Real-time PCR Master Mix (TOYOBOCO, LTD, Osaka, Japan) in a Roche LightCycler 480II (Roche, Basel, Sweden), according to the manufacturer’s instructions. Real-time PCR was performed at 95 °C for 10 min, followed by 45 cycles of 95 °C for 15 s, 60 °C for 60 s, and 72 °C for 30 s. β-Actin was used as an internal reference to normalize target gene expression. The 2^−∆∆*C*t^ method was used to calculate relative changes in gene expression between control and experimental groups. Finally, PCR products were gel extracted and subjected to Sanger sequencing.

## 3. Results

### 3.1. Overview of circRNA Profiles in Polytocous and Monotocous Sheep Uteri

**Figure 1:**
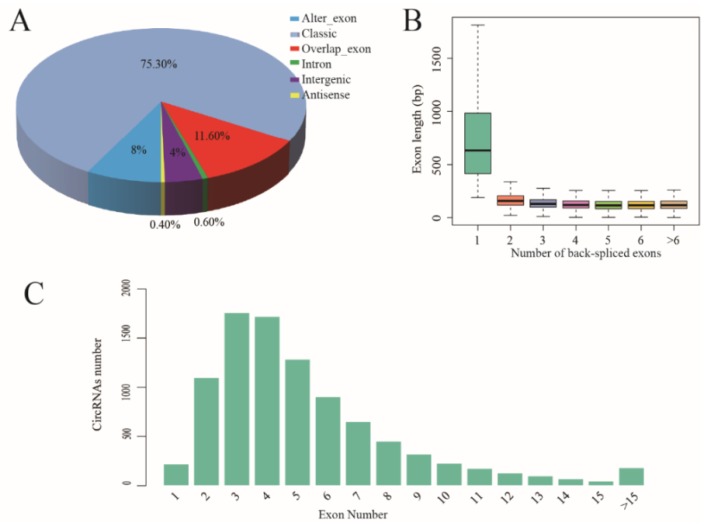
General characteristics of circRNAs in the sheep uteri. (**A**) Percentages of the six types of circRNAs. (**B**) Exon length distribution of cricRNAs. (**C**) Number of exons per transcript of sheep circRNA.

To identify differentially expressed circRNAs during follicular and lutein phases in the uteri of polytocous and monotocous Small Tail Han sheep, we used deep sequencing to analyze circRNAs. We obtained 371,999,010 and 362,973,390 clean reads from polytocous and monotocous sheep during the follicular stage, and 380,416,106 and 373,756,026 clean reads from polytocous and monotocous sheep during the luteal phase, respectively. A total of 32,687 candidate circRNAs were identified by at least one read spanning a head-to-tail splice junction, according to the splice site information of the circular RNA and the relative position of the gene structure. These circRNAs were divided into six types, alter_exon (8.0%), classic (75.3%), intron (0.6%), overlap_exon (11.6%), intergenic (4.0%) and antisense (0.4%) (Figure 1A). When the circular RNA consisted of one exon, the length of the exon was significantly longer than that of the circular RNA consisting of multiple exons (Figure 1B). Many circRNAs had only three or four exons (Figure 1C).

### 3.2. Differential Expression Analysis of circRNAs

**Figure 2:**
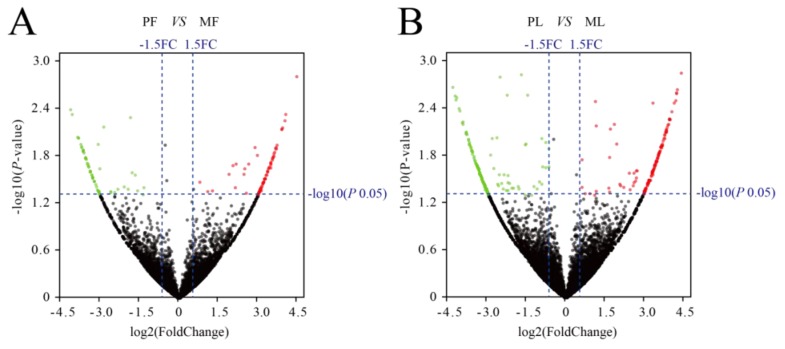
Analysis of differentially expressed circRNAs. (**A**) Differentially expressed circRNAs in the follicular phase. (**B**) Differentially expressed circRNAs in the luteal phase. Red, green, and gray dots in the graph represent transcripts that were significantly up-regulated, down-regulated, or unchanged respectively, between polytocous and monotocous sheep.

Differentially expressed circRNAs in the uterine tissues of polytocous and monotocous sheep were identified by DEseq2, based on a fold change of ≥1.5 and a *p*-value < 0.05. In the follicular phase, 147 DE-circRNAs were found in PF and MF groups, including 80 up-regulated and 67 down-regulated circRNAs (Figure 2A, Table S1). In the lutein phase, 364 DE-circRNAs were found in PL and ML groups, including 178 up-regulated and 186 down-regulated circRNAs (Figure 2B, Table S1).

### 3.3. Gene Ontology (GO) and KEGG Pathway Enrichment Analyses

**Figure 3:**
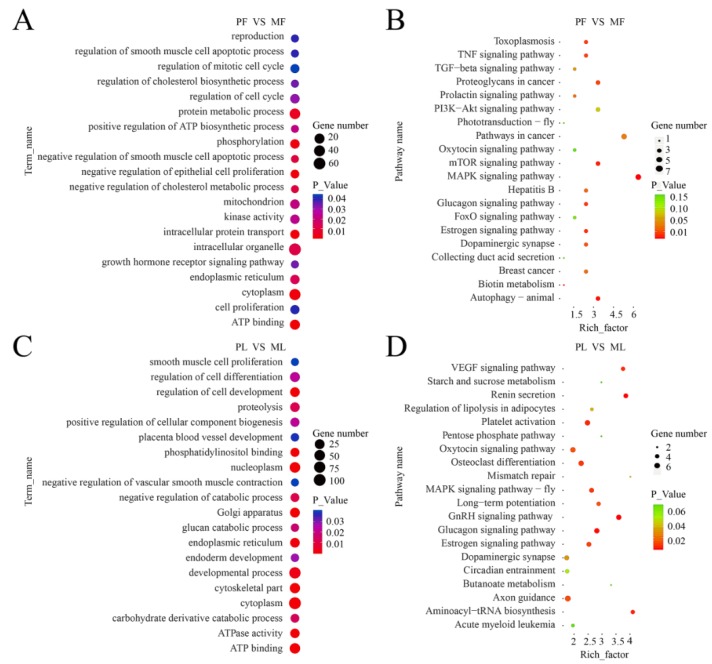
GO and KEGG analyses of differentially expressed circRNA host genes. (**A**) GO functional analysis of differentially expressed circRNA host genes in the follicular phase. (**B**) KEGG analysis of differentially expressed circRNA host genes in the follicular phase. (**C**) GO functional analysis of differentially expressed circRNA host genes in the luteal phase. (**D**) KEGG analysis of differentially expressed circRNA host genes in the luteal phase. The longitudinal and horizontal axes represent the enrichment pathways and rich factor (amount of differentially expressed genes enriched in the pathway/amount of all genes in the background gene set) of these pathways, respectively. The spot size represents the number of differentially expressed genes enriched in each pathway, and the color of the spot represents the *p*-value of each pathway.

In the follicular phase, GO terms analyses of the 147 DE-circRNA host genes revealed significantly enriched terms (*p* < 0.05) in the categories of biological process, molecular function, and cellular components (Table S2). Figure 3A shows the significantly enriched biological processes such as reproduction, regulation of a cell cycle, cell proliferation and growth hormone receptor signaling pathway. A total of 154 terms were enriched in the KEGG pathway analysis in which the estrogen signaling pathway, biotin metabolism, prolactin signaling pathway, TGF-beta signaling pathway and oxytocin signaling pathway were related to reproduction (Figure 3B, Table S3).

In the luteal phase, GO terms analyses of the 364 DE-circRNA host genes revealed significantly enriched terms (*p* < 0.05) in the categories of biological process, molecular function, and cellular components (Table S2). Figure 3C shows the significantly enriched biological processes such as the developmental process, regulation of cell development, carbohydrate derivative catabolic process, placenta blood vessel development, and glucan catabolic process. A total of 228 terms were enriched in the KEGG pathway analysis in which the GnRH signaling pathway, VEGF signaling pathway, estrogen signaling pathway, oxytocin signaling pathway, pentose phosphate pathway, and starch and sucrose metabolism were related to reproduction and energy metabolism (Figure 3D, Table S3).

### 3.4. Target miRNAs of Differentially Expressed circRNAs in PG and MG

**Figure 4:**
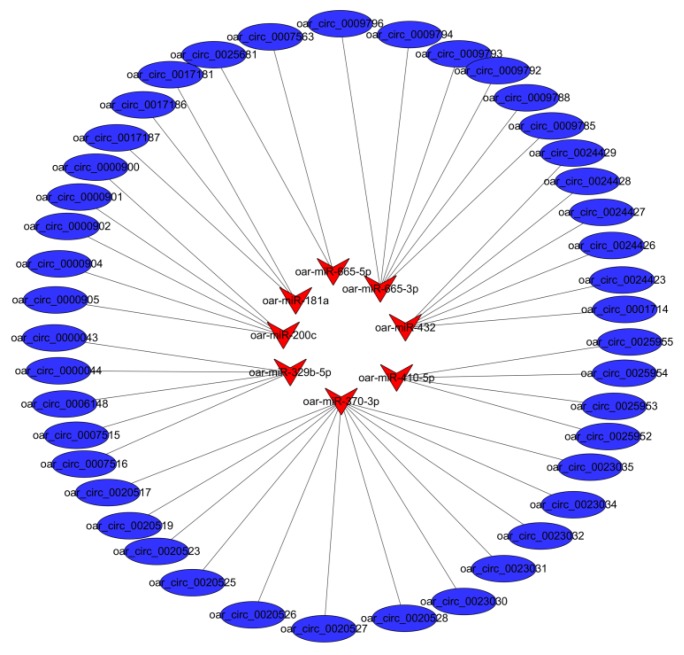
Regulatory networks of circRNA-miRNA interactions in the follicular phase. Triangular nodes represent miRNA, and circular nodes represent circRNA.

**Figure 5:**
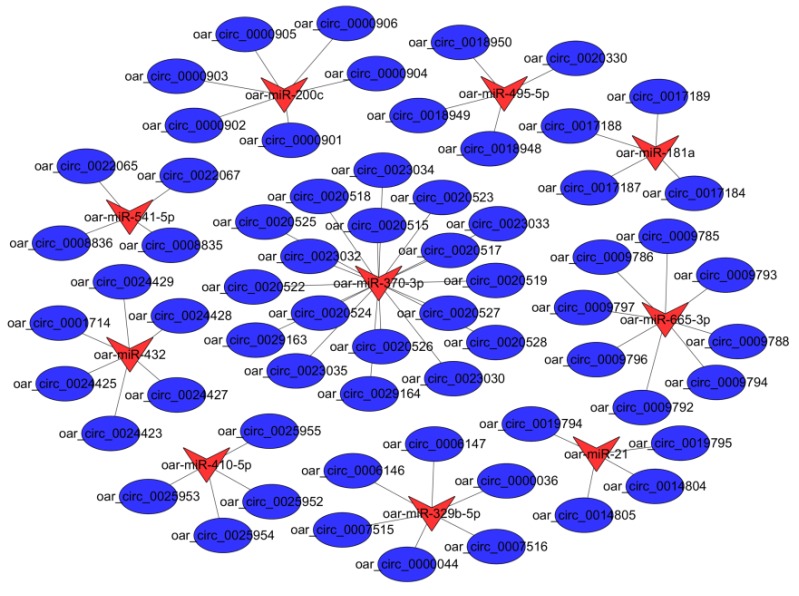
Regulatory networks of circRNA-miRNA interactions in the luteal phase. Triangular nodes represent miRNA, and circular nodes represent circRNA.

A total of 100 differently expressed circRNAs targeting 26 miRNAs were found. Subsequently, we analyzed the interaction between circRNAs and predicted target miRNAs. The resulting circRNA-miRNA association network provided nodes and linkages between circRNAs and their target miRNAs (Table S4). In the follicular phase, a comparison of the circRNA-miRNA networks of PG and MG groups revealed eight miRNAs interacting with 43 differentially expressed circRNAs associated with uterine functions, and oar-miR-370-3p targeted up to 12 cirRNAs (Figure 4). In the luteal phase, a comparison of the circRNA-miRNA networks of PG and MG groups revealed 10 miRNAs interacting with 64 differentially expressed circRNAs associated with uterine functions, and oar-miR-665-3p and oar-miR-370-3p targeted up to 10 and 18 cirRNAs, respectively (Figure 5).

### 3.5. Validation of circRNA Expression

**Figure 6:**
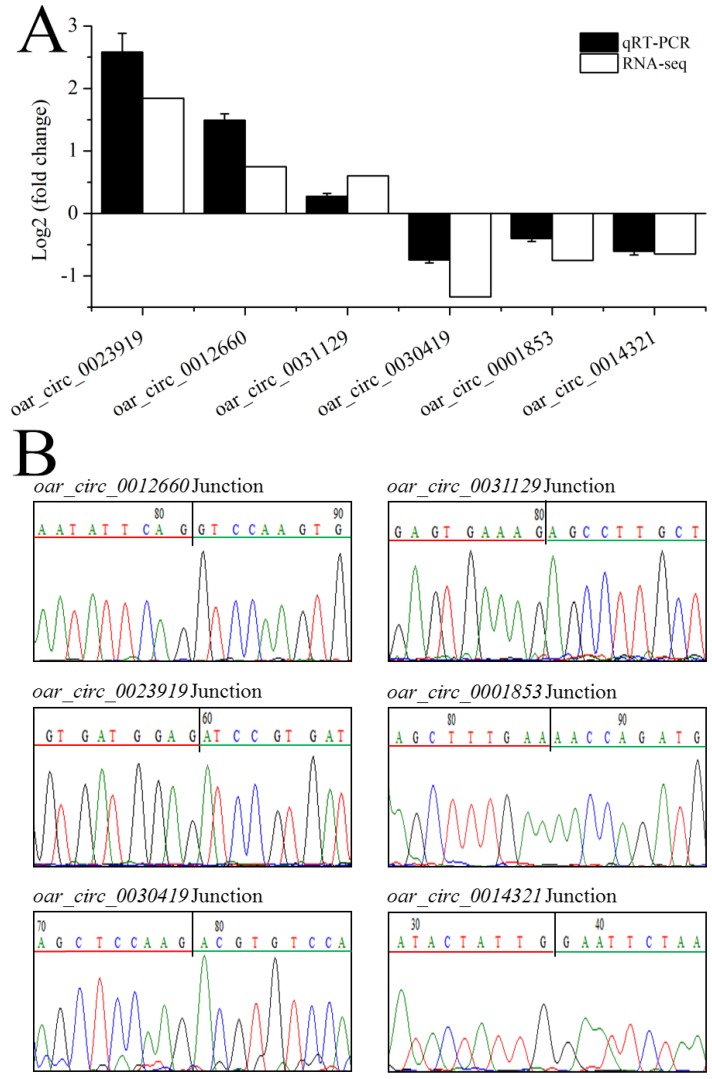
Validation of circRNA expression by qRT-PCR and Sanger sequencing. (**A**) qRT-PCR verification of differentially expressed circRNAs. (**B**) Back-spliced junctions of the six randomly selected circRNAs was confirmed by Sanger sequencing. The black vertical line represents the back-spliced junction sites. Green and red horizontal lines indicate the 5’ and 3’ ends of the circRNA sequence, respectively.

We selected six differentially expressed circRNAs for quantitative PCR (qPCR) to confirm the sequencing data (Figure 6A) and verify the specificity by Sanger sequencing (Figure 6B). The expression levels of these circRNAs detected by real-time PCR were consistent with those obtained by sequencing, confirming the reliability of our sequencing results.

## 4. Discussion

In previous studies, circRNAs had been considered as RNA splicing errors [46]. Recent studies have demonstrated that circRNA plays an important role in the biology and developmental processes of sheep [32,35]. However, uterine circRNAs in sheep have received little attention compared with other tissue non-coding RNAs [33,34]. Here, we identified 32,687 circRNAs in the uterus ofpolytocous and monotocous Small Tail Han sheep. Many circRNAs had only three or four exons, which is similar to previous findings in sheep skeletal muscle [34]. These observations in the uterus of Small Tail Han sheep combined with previous findings suggest that the exon number is a universal feature of the uterus.

Research regarding the functional roles of circRNAs in this field is still at its infancy [47]. Presently, there are reports about the expression and potential biological functions of circRNAs in reproductive organs in *xenopus* tropicalis [48], mice [49,50] and humans [51,52,53]. These early studies laid the foundation for further research. In mice, 2891 circRNAs have been found in oocytes and early embryos, of which about 91% consist of multiple exons. In addition, host genes producing these circRNAs revealed that circRNAs in mouse early embryos may be responsible for cell division and DNA repair [48]. Eleven circRNAs were significantly downregulated, and 46 circRNAs are significantly upregulated in advanced age female granulosa cells [54]. In this study, 147 and 364 circRNAs were differentially expressed in polytocous and monotocous groups in the follicular phase and luteal phases, respectively. Therefore, we predicted that circRNAs might play important roles in the prolificacy of sheep. The apparent regulation of circRNA appears to be a common phenomenon.

The response to prolificacy in animals is a complicated process, involving several genes and metabolic networks. Hormone synthesis and nutrients are important factors [55,56,57]. In our study, four host genes, including *AKT3*, *ITPR1*, *ITPR2*, and *PRKACA*, are involved in reproduction-related pathways such as GnRH, estrogen, oxytocin and TGF-beta signaling pathways. Studies have shown that GnRH and GnRHR are expressed in human endometrial epithelial cells and stromal cells [58,59]. During the follicular phase, the GnRH/GnRHR system induces a rapid increase in the plasma E2 concentration, leading to the LH preovulatory surge [60]. During the luteal phase, FSH gradually increases with decreasing LH [61]. The GnRH/GnRHR system might play a key role in promoting trophoblast invasion of the maternal endometrium during embryo implantation [62,63]. Estrogen is the major regulator of placental growth and uterine functions in sheep [64]. In the luteal phase, estrogen plays a regulatory role in vascular functions of the uterus and placenta, such as regulation of blood flow, vascular tone, promoting angiogenesis, and vascular remodeling [65,66,67]. One of the most common effects of oxytocin (OT) is the induction of uterine contractions and childbirth [68]. OT stimulates uterine activity of estrus sows and promotes uterine contractions and muscle hyperplasia [69,70]. It is well known that the TGF-β signaling pathway is essential for the normal follicular development and functions of ovarian cells [71,72,73].

In addition, host genes *PGM2* and *UGP2* expressed in the luteal phase are involved in energy metabolism-related pathways such as the pentose phosphate pathway, galactose metabolism, and starch and sucrose metabolism. As the animal transitions from ovulation to the seventh day of the estrous cycle, the uterine metabolome also changes. Amino acids, benzoic acid, lipid molecules, carbohydrates, purines, pyrimidines, vitamins, and other intermediate and secondary metabolites reach maximum intensities on either day five or seven relative to ovulation [74,75]. In the present study, the results of the KEGG pathway analysis showed that circRNAs were associated with hormone synthesis and energy metabolism, suggesting their potential effects on reproduction by regulating uterine hormone synthesis and energy metabolism, although the specific mechanism remains to be elucidated.

CircRNA can bind to miRNAs and compete with endogenous RNA [76]. Therefore, some circRNAs may inhibit or relieve repression of miRNA for translation [77]. In our study, 100 differently expressed circRNAs targeting 26 miRNAs were found. We deduced that various circRNAs containing common miRNA-binding sites might act as miRNA sponges to regulate the response of the sheep uterus to prolificacy.

## 5. Conclusions

We examined the abundance, genomic characteristics, and length distribution of uterine circRNAs, which lay the foundation for the study of uterine circRNAs in Small Tail Han sheep. Meanwhile, we find that several circRNAs take part in the process of development and uterine growth by KEGG pathway analysis. Function prediction indicates that circRNAs play an important role in the reproduction. Our study provides a valuable resource for circRNAs biology, as well as contributes to understanding circRNAs function in growth and development of uterine.

## Figures and Tables

**Table 1:** Details of primer sequences used for qRT-PCR and expected product sizes of circRNAs.

Gene Name	Primer Sequence (5′–3′)	Product Size (bp)
oar_circ_0030419	F: CCCATCCCCGGCACGTCCA R: GTCCCGTCCTCACTGCACTCG	120
oar_circ_0012660	F: AAACCTGGCACGTACGCAGA R: AGGTTGACGGCATACATCAGC	199
oar_circ_0023919	F: CTTCCTTCAGACAGAATGCAC R: CGAGCTCTCCAATATTGTCAC	169
oar_circ_0031129	F: GAGTGAATAAAGATGTCATCCGA R: ACCAGAATATTCAGAAATGTGCC	147
oar_circ_0014321	F: GAAACTGCACACAGATAACCA R: ACATGCTGGATCTAAAACCAC	160
oar_circ_0001853	F: AAGTCACTTAAAACGCACCT R: TAAATTACTGTTCTCCGCTTC	180
β-Actin	F:CCAACCGTGAGAAGATGACC R:CCCGAGGCGTACAGGGACAG	97

## Supplementary Materials

The following are available online at https://www.mdpi.com/2076-2615/9/10/797/s1, Table S1: Differentially expressed circRNAs between PG and MG, Table S2: Enriched GO terms of host genes of differentially expressed circRNAs, Table S3: Enriched KEGG pathways of host genes of differentially expressed circRNAs, Table S4: List of circRNA-miRNA pairs to construct the interaction network.

## References

[1] Miao X., Luo Q., Zhao H., Qin X. (2016). Ovarian proteomic study reveals the possible molecular mechanism for hyperprolificacy of Small Tail Han sheep. Sci. Rep..

[2] Davis G.H., Montgomery G.W., Allison A.J., Kelly R.W., Bray A.R. (1982). Segregation of a major gene influencing fecundity in progeny of Booroola sheep. N. Z. J. Agric..

[3] Fogarty N.M. (2009). A review of the effects of the Booroola gene (FecB) on sheep production. Small Rumin. Res..

[4] Wilson T., Wu X.Y., Juenge J.L., Ross I.K., Lumsden J.M., Lord E.A., Dodds K.G., Walling G.A., McEwan J.C., Connell A.R. (2001). Highly prolific Booroola sheep have a mutation in the intracellular kinase domain of bone morphogenetic protein IB receptor (ALK-6) that is expressed in both oocytes and granulosa cells. Biol. Reprod..

[5] Souza C.J., MacDougall C., Campbell B.K., McNeilly A.S., Baird D.T. (2001). The Booroola (FecB) phenotype is associated with a mutation in the bone morphogenetic receptor type 1 B (BMPR1B) gene. J. Endocrinol..

[6] Mulsant P., Lecerf F., Fabre S., Schibler L., Monget P., Lanneluc I., Pisselet C., Riquet J., Monniaux D., Callebaut I. (2001). Mutation in bone morphogenetic protein receptor-IB is associated with increased ovulation rate in Booroola Mérino ewes. Proc. Natl. Acad. Sci. USA.

[7] McBride D., Carre W., Sontakke S.D., Hogg C.O., Law A., Donadeu F.X., Clinton M. (2012). Identification of miRNAs associated with the follicular-luteal transition in the ruminant ovary. Reproduction.

[8] Mihm M., Gangooly S., Muttukrishna S. (2011). The normal menstrual cycle in women. Anim. Reprod. Sci..

[9] Miao X., Luo Q., Zhao H., Qin X. (2016). Ovarian transcriptomic study reveals the differential regulation of miRNAs and lncRNAs related to fecundity in different sheep. Sci. Rep..

[10] Miao X., Luo Q., Qin X. (2016). Genome-wide transcriptome analysis in the ovaries of two goats identifies differentially expressed genes related to fecundity. Gene.

[11] Sharma A., Kumar P. (2012). Understanding implantation window, a crucial phenomenon. J. Hum. Reprod. Sci..

[12] Kao L.C., Tulac S., Lobo S., Imani B., Yang J.P., Germeyer A., Osteen K., Taylor R.N., Lessey B.A., Giudice L.C. (2002). Global gene profiling in human endometrium during the window of implantation. Endocrinology.

[13] Strassmann B.I. (1996). The evolution of endometrial cycles and menstruation. Q. Rev. Biol..

[14] Groebner A.E., Rubio-Aliaga I., Schulke K., Reichenbach H.D., Daniel H., Wolf E., Meyer H.H., Ulbrich S.E. (2011). Increase of essential amino acids in the bovine uterine lumen during preimplantation development. Reproduction.

[15] Harris S.E., Gopichandran N., Picton H.M., Leese H.J., Orsi N.M. (2005). Nutrient concentrations in murine follicular fluid and the female reproductive tract. Theriogenology.

[16] Bazer F.W. (1975). Uterine protein secretions: Relationship to development of the conceptus. J. Anim. Sci..

[17] Gao H., Wu G., Spencer T.E., Johnson G.A., Li X., Bazer F.W. (2009). Select nutrients in the ovine uterine lumen. I. Amino acids, glucose, and ions in uterine lumenal flushings of cyclic and pregnant ewes. Biol. Reprod..

[18] Paria B.C., Reese J., Das S.K., Dey S.K. (2002). Deciphering the cross-talk of implantation: Advances and challenges. Science.

[19] Lim H., Ma L., Ma W.G., Maas R.L., Dey S.K. (1999). Hoxa-10 regulates uterine stromal cell responsiveness to progesterone during implantation and decidualization in the mouse. Mol. Endocrinol..

[20] Lijie S., Ruize L., Wei C., Mengjin Z., Xiaoping L., Shuhong Z., Mei Y. (2014). Expression patterns of microRNAs in porcine endometrium and their potential roles in embryo implantation and placentation. PLoS ONE.

[21] Xia H.F., Jin X.H., Cao Z.F., Hu Y., Ma X. (2014). MicroRNA expression and regulation in the uterus during embryo implantation in rat. FEBS J..

[22] Salzman J., Gawad C., Wang P.L., Lacayo N., Brown P.O. (2012). Circular RNAs are the predominant transcript isoform from hundreds of human genes in diverse cell types. PLoS ONE.

[23] Sanger H.L., Klotz G., Riesner D., Gross H.J., Kleinschmidt A.K. (1976). Viroids are single-stranded covalently closed circular RNA molecules existing as highly base-paired rod-like structures. Proc. Natl. Acad. Sci. USA.

[24] Jeck W.R., Sharpless N.E. (2014). Detecting and characterizing circular RNAs. Nat. Biotechnol..

[25] Bhosale R., Boudolf V., Cuevas F., Lu R., Eekhout T., Hu Z., van Isterdael G., Lambert G.M., Xu F., Nowack M.K. (2018). A spatiotemporal DNA endoploidy map of the Arabidopsis root reveals roles for the endocycle in root development and stress adaptation. Plant Cell.

[26] Cai Y., Cai X., Wang Q., Wang P., Zhang Y., Cai C., Xu Y., Wang K., Zhou Z., Wang C. (2019). Genome sequencing of the Australian wild diploid species *Gossypium australe* highlights disease resistance and delayed gland morphogenesis. Plant Biotechnol. J..

[27] La Y., Tang J., He X., Di R., Wang X., Liu Q., Zhang L., Zhang X., Zhang J., Hu W. (2019). Identification and characterization of mRNAs and lncRNAs in the uterus of polytocous and monotocous Small Tail Han sheep (Ovis aries). Peer J..

[28] Veno M.T., Hansen T.B., Veno S.T., Clausen B.H., Grebing M., Finsen B., Holm I.E., Kjems J. (2015). Spatio-temporal regulation of circular RNA expression during porcine embryonic brain development. Genome Biol..

[29] Hansen T.B., Jensen T.I., Clausen B.H., Bramsen J.B., Bente F., Damgaard C.K., Kjems J. (2013). Natural RNA circles function as efficient microRNA sponges. Nature.

[30] Memczak S., Jens M., Elefsinioti A., Torti F., Krueger J., Rybak A., Maier L., Mackowiak S.D., Gregersen L.H., Munschauer M. (2013). Circular RNAs are a large class of animal RNAs with regulatory potency. Nature.

[31] Zhang L., Liu X., Che S., Cui J., Liu Y., An X., Cao B., Song Y. (2018). CircRNA-9119 regulates the expression of prostaglandin-endoperoxide synthase 2 (PTGS2) by sponging miR-26a in the endometrial epithelial cells of dairy goat. Reprod. Fertil. Dev..

[32] Li C., Li X., Ma Q., Zhang X., Cao Y., Yao Y., You S., Wang D., Quan R., Hou X. (2017). Genome-wide analysis of circular RNAs in prenatal and postnatal pituitary glands of sheep. Sci. Rep..

[33] Li C., Li X., Yao Y., Ma Q., Ni W., Zhang X., Cao Y., Hazi W., Wang D., Quan R. (2017). Genome-wide analysis of circular RNAs in prenatal and postnatal muscle of sheep. Oncotarget.

[34] Cao Y., You S., Yao Y., Liu Z.J., Hazi W., Li C.Y., Zhang X.Y., Hou X.X., Wei J.C., Li X.Y. (2018). Expression profiles of circular RNAs in sheep skeletal muscle. Asian Australas. J. Anim. Sci..

[35] Jin C., Bao J., Wang Y., Chen W., Zou S., Wu T., Wang L., Lv X., Gao W., Wang B. (2018). Changes in circRNA expression profiles related to the antagonistic effects of *Escherichia coli* F17 in lamb spleens. Sci. Rep..

[36] Long L., Yang W.W., Liao P., Guo Y.W., Kumar A., Gao W. (2019). Transcriptome analysis reveals differentially expressed ERF transcription factors associated with salt response in cotton. Plant Sci..

[37] Wang D., Yang C., Long D., Zhu J., Wang J., Zhang S. (2015). Comparative transcriptome analyses of drought-resistant and - susceptible *Brassica napus* L. and development of EST-SSR markers by RNA-Seq. J. Plant Biol..

[38] Wang P., Yang C., Chen H., Luo L., Leng Q., Li S., Han Z., Li X., Song C., Zhang X. (2018). Exploring transcription factors reveals crucial members and regulatory networks involved in different abiotic stresses in *Brassica napus* L.. BMC Plant Biol..

[39] Zhang Z., Tang J., Di R., Liu Q., Wang X., Gan S., Zhang X., Zhang J., Hu W., Chu M. (2019). Comparative transcriptomics reveal key sheep (Ovis aries) hypothalamus lncRNAs that affect reproduction. Animals.

[40] Gao Y., Wang J., Zhao F. (2015). CIRI: An efficient and unbiased algorithm for de novo circular RNA identification. Genome Biol..

[41] Houtgast E.J., Sima V.M., Bertels K., Al-Ars Z. (2018). Hardware acceleration of BWA-MEM genomic short read mapping for longer read lengths. Comput. Biol. Chem..

[42] Li Y., Zheng Q., Bao C., Li S., Guo W., Zhao J., Chen D., Gu J., He X., Huang S. (2015). Circular RNA is enriched and stable in exosomes: A promising biomarker for cancer diagnosis. Cell Res..

[43] Love M.I., Huber W., Anders S. (2014). Moderated estimation of fold change and dispersion for RNA-seq data with DESeq2. Genome Biol..

[44] Ashburner M., Ball C.A., Blake J.A., Botstein D., Butler H., Cherry J.M., Davis A.P., Dolinski K., Dwight S.S., Eppig J.T. (2000). Gene ontology: Tool for the unification of biology. Nat. Genet..

[45] Pasquinelli A.E. (2012). MicroRNAs and their targets: Recognition, regulation and an emerging reciprocal relationship. Nat. Rev. Genet..

[46] Zhao W., Cheng Y., Zhang C., You Q., Shen X., Guo W., Jiao Y. (2017). Genome-wide identification and characterization of circular RNAs by high throughput sequencing in soybean. Sci. Rep..

[47] Quan G., Li J. (2018). Circular RNAs: Biogenesis, expression and their potential roles in reproduction. J. Ovarian Res..

[48] Talhouarne G.J., Gall J.G. (2014). Lariat intronic RNAs in the cytoplasm of Xenopus tropicalis oocytes. RNA.

[49] Fan X., Zhang X., Wu X., Guo H., Hu Y., Tang F., Huang Y. (2015). Single-cell RNA-seq transcriptome analysis of linear and circular RNAs in mouse preimplantation embryos. Genome Biol..

[50] Lin X., Han M., Cheng L., Chen J., Zhang Z., Shen T., Wang M., Wen B., Ni T., Han C. (2016). Expression dynamics, relationships, and transcriptional regulations of diverse transcripts in mouse spermatogenic cells. RNA Biol..

[51] Dong W.W., Li H.M., Qing X.R., Huang D.H., Li H.G. (2016). Identification and characterization of human testis derived circular RNAs and their existence in seminal plasma. Sci. Rep..

[52] Qian Y., Lu Y., Rui C., Qian Y., Cai M., Jia R. (2016). Potential Significance of Circular RNA in Human Placental Tissue for Patients with Preeclampsia. Cell Physiol. Biochem..

[53] Dang Y., Yan L., Hu B., Fan X., Ren Y., Li R., Lian Y., Yan J., Li Q., Zhang Y. (2016). Tracing the expression of circular RNAs in human pre-implantation embryos. Genome Biol..

[54] Cheng J., Huang J., Yuan S., Zhou S., Yan W., Shen W., Chen Y., Xia X., Luo A., Zhu D. (2017). Circular RNA expression profiling of human granulosa cells during maternal aging reveals novel transcripts associated with assisted reproductive technology outcomes. PLoS ONE.

[55] Rhinehart E.M. (2016). Mechanisms linking energy balance and reproduction: Impact of prenatal environment. Horm. Mol. Biol. Clin. Investig..

[56] Härter C.J., Lima L.D., Hgo S., Castagnino D.S., Rivera A.R., Resende K.T., Iama T. (2017). Energy and protein requirements for maintenance of dairy goats during pregnancy and their efficiencies of use. J. Anim. Sci..

[57] Schneider J.E. (2004). Energy balance and reproduction. Physiol. Behav..

[58] Raga F., Casan E.M., Kruessel J.S., Wen Y., Huang H.Y., Nezhat C., Polan M.L. (1998). Quantitative gonadotropin-releasing hormone gene expression and immunohistochemical localization in human endometrium throughout the menstrual cycle. Biol. Reprod..

[59] Limonta P., Marelli M.M., Moretti R., Marzagalli M., Fontana F., Maggi R. (2018). GnRH in the human female reproductive axis. Vitam. Horm..

[60] Moenter S.M., Caraty A., Locatelli A., Karsch F.J. (1991). Pattern of gonadotropin-releasing hormone (GnRH) secretion leading up to ovulation in the ewe: Existence of a preovulatory GnRH surge. Endocrinology.

[61] Rance N.E. (2009). Menopause and the human hypothalamus: Evidence for the role of kisspeptin/neurokinin B neurons in the regulation of estrogen negative feedback. Peptides.

[62] Chou C.S., MacCalman C.D., Leung P.C. (2003). Differential effects of gonadotropin-releasing hormone I and II on the urokinase-type plasminogen activator/plasminogen activator inhibitor system in human decidual stromal cells in vitro. J. Clin. Endocrinol. Metab..

[63] Wu H.M., Huang H.Y., Lee C.L., Soong Y.K., Leung P.C., Wang H.S. (2015). Gonadotropin-releasing hormone type II (GnRH-II) agonist regulates the motility of human decidual endometrial stromal cells: Possible effect on embryo implantation and pregnancy. Biol. Reprod..

[64] Grazul-Bilska A.T., Bairagi S., Kraisoon A., Dorsam S.T., Reyaz A., Navanukraw C., Borowicz P.P., Reynolds L.P. (2019). Placental development during early pregnancy in sheep: Nuclear estrogen and progesterone receptor mRNA expression in the utero-placental compartments. Domest. Anim. Endocrinol..

[65] Boos A., Kohtes J., Stelljes A., Zerbe H., Thole H.H. (2000). Immunohistochemical assessment of progesterone, oestrogen and glucocorticoid receptors in bovine placentomes during pregnancy, induced parturition, and after birth with or without retention of fetal membranes. J. Reprod. Fertil..

[66] Pastore M.B., Jobe S.O., Jayanth R., Magness R.R. (2012). Estrogen receptor-α and estrogen receptor-β in the uterine vascular endothelium during pregnancy: Functional implications for regulating uterine blood flow. Semin. Reprod. Med..

[67] Wu W.X., Owiny J., Zhang Q., Ma X.H., Nathanielsz P.W. (1996). Regulation of the estrogen receptor and its messenger ribonucleic acid in the ovariectomized sheep myometrium and endometrium: The role of estradiol and progesterone. Biol. Reprod..

[68] Kim S.H., Pohl O., Chollet A., Gotteland J.P., Fairhurst A.D., Bennett P.R., Terzidou V. (2017). Differential Effects of Oxytocin Receptor Antagonists, Atosiban and Nolasiban, on Oxytocin Receptor-Mediated Signaling in Human Amnion and Myometrium. Mol. Pharmacol..

[69] Langendijk P., Bouwman E.G., Schams D., Soede N.M., Kemp B. (2003). Effects of different sexual stimuli on oxytocin release, uterine activity and receptive behavior in estrous sows. Theriogenology.

[70] Domino M., Pawlinski B., Gajewska M., Jasinski T., Sady M., Gajewski Z. (2018). Uterine EMG activity in the non-pregnant sow during estrous cycle. BMC Vet. Res..

[71] Twombly V., Blackman R.K., Jin H., Graff J.M., Padgett R.W., Gelbart W.M. (1996). The TGF-beta signaling pathway is essential for Drosophila oogenesis. Development.

[72] Elvin J.A., Yan C., Matzuk M.M. (2000). Oocyte-expressed TGF-beta superfamily members in female fertility. Mol. Cell Endocrinol..

[73] Miao X., Luo Q., Zhao H., Qin X. (2016). Co-expression analysis and identification of fecundity-related long non-coding RNAs in sheep ovaries. Sci. Rep..

[74] Tribulo P., Balzano-Nogueira L., Conesa A., Siqueira L.G., Hansen P.J. (2018). Changes in the uterine metabolome of the cow during the first 7 days after estrus. Mol. Reprod. Dev..

[75] Romero J.J., Liebig B.E., Broeckling C.D., Prenni J.E., Hansen T.R. (2017). Pregnancy-induced changes in metabolome and proteome in ovine uterine flushings. Biol. Reprod..

[76] Wang J., Lin J., Wang H., Li X., Yang Q., Li H., Chang Y. (2018). Identification and characterization of circRNAs in Pyrus betulifolia Bunge under drought stress. PLoS ONE.

[77] Gao Y., Wu M., Fan Y., Li S., Lai Z., Huang Y., Lan X., Lei C., Chen H., Dang R. (2018). Identification and characterization of circular RNAs in Qinchuan cattle testis. R. Soc. Open Sci..

