# Supplementary material for: Differential Expression of Circular RNAs in Polytocous and Monotocous Uterus during the Reproductive Cycle of Sheep
Source: Animals (Basel). 2019 Oct 14;9(10):797. doi: 10.3390/ani9100797 (PMC6827132; doi:10.3390/ani9100797)
Supplement: Supplementary file 1 [file animals-09-00797-s001.zip › Supplemental File legend.docx]

**The raw data were applied to the screening and functional analysis of differentially expressed circRNAs in PG and MG in the follicular and luteal phases, respectively.**

Table S1 Differentially expressed circRNAs between PG and MG, Table S2 Enriched GO terms of host genes of differentially expressed circRNAs, Table S3 Enriched KEGG pathways of host genes of differentially expressed circRNAs, Table S4 List of circRNA-miRNA pairs to construct the interaction network.
